# Providing care to marginalised communities: a qualitative study of community pharmacy teams

**DOI:** 10.3399/BJGP.2023.0267

**Published:** 2023-12-29

**Authors:** Helen Gibson, Caroline Sanders, Thomas Blakeman, Darren M Ashcroft, Nina Fudge, Kelly Howells

**Affiliations:** National Institute for Health and Care Research (NIHR) School for Primary Care Research, School of Health Sciences, Faculty of Biology, Medicine and Health, The University of Manchester; NIHR Greater Manchester Patient Safety Research Collaboration, The University of Manchester, Manchester, UK.; National Institute for Health and Care Research (NIHR) School for Primary Care Research, School of Health Sciences, Faculty of Biology, Medicine and Health, The University of Manchester; NIHR Greater Manchester Patient Safety Research Collaboration, The University of Manchester, Manchester, UK.; National Institute for Health and Care Research (NIHR) School for Primary Care Research, School of Health Sciences, Faculty of Biology, Medicine and Health, The University of Manchester; NIHR Greater Manchester Patient Safety Research Collaboration, The University of Manchester, Manchester, UK.; NIHR School for Primary Care Research, School of Health Sciences, Faculty of Biology, Medicine and Health, The University of Manchester; NIHR Greater Manchester Patient Safety Research Collaboration, The University of Manchester; Division of Pharmacy and Optometry, School of Health Sciences, Faculty of Biology, Medicine and Health, The University of Manchester, Manchester, UK.; Wolfson Institute of Population Health, Queen Mary University of London, London.; National Institute for Health and Care Research (NIHR) School for Primary Care Research, School of Health Sciences, Faculty of Biology, Medicine and Health, The University of Manchester; NIHR Greater Manchester Patient Safety Research Collaboration, The University of Manchester, Manchester, UK.

**Keywords:** community pharmacy services, healthcare inequalities, medically underserved populations, primary health care, qualitative research

## Abstract

**Background:**

Health inequalities in the UK are widening, particularly since the COVID-19 pandemic. Community pharmacies are the most visited healthcare provider in England and are ideally placed to provide and facilitate access to care for those most disadvantaged.

**Aim:**

To explore the experiences and needs of community pharmacy teams in providing care for marginalised groups and how this has changed since the COVID-19 pandemic.

**Design and setting:**

A qualitative study in community pharmacy and across primary care.

**Method:**

Semi-structured interviews were undertaken with members of community pharmacy teams, primary care network (PCN) pharmacists, GPs, and nurses in the North of England.

**Results:**

In total, 31 individuals participated in an interview (26 pharmacy staff, three GPs, and two nurses). Most participants acknowledged that their pharmacy had become busier since COVID-19 because of increased footfall compounded by patient difficulties in navigating remote digital systems. Few participants had received any formal training on working with marginalised communities; however, organisational barriers (such as lack of access to translation facilities) combined with interorganisational barriers (such as lack of integrated care) made it more difficult to provide care for some marginalised groups. Despite this, the continuity of care provided by many pharmacies was viewed as an important factor in enabling marginalised groups to access and receive care.

**Conclusion:**

There are opportunities to better utilise the skills of community pharmacy teams. Resources, such as access to translation services, and interventions to enable better communication between community pharmacy teams and other primary care services, such as general practice, are essential.

## Introduction

Health inequalities in the UK are widening, with recent data showing that the most deprived areas of society have been disproportionately affected by the pandemic, particularly in the North of England.^1^^–^^3^ Marginalised and vulnerable groups can include people experiencing homelessness, drug and alcohol dependence, vulnerable migrants, Gypsy, Roma and Traveller communities, sex workers, people in contact with the justice system, victims of modern slavery, and other socially excluded groups.^4^ People from marginalised communities are more likely to have at least one chronic, long-term condition and lower life expectancy than the general population.^2^

The NHS Long Term Plan seeks to close the inequality gap, and recommends that primary care services adapt and collaborate to prioritise the needs of disadvantaged groups.^5^ Community pharmacies are the most visited healthcare provider in England with approximately 1.6 million visits a day.^6^^,^^7^ They do not require an appointment, often have long opening hours, and are generally accessible by foot for the majority of the UK population.^7^^,^^8^ Community pharmacies are described as *‘socially inclusive’* as they provide *‘a convenient and less formal environment for people who cannot easily access or do not choose to access other kinds of health services’*.^9^

The potential role of community pharmacists in providing health services to hard-to-reach populations has been recognised for some time.^7^^,^^10^ Recent expanded roles in community pharmacy include health prevention (for example, weight management, smoking cessation, vaccinations, and needle exchange) and health screening (for example, for diabetes and chlamydia). Numerous studies have noted the potential of community pharmacists to prevent suicide and self-harm,^6^ provide substance use support,^11^ and deliver care for people experiencing homelessness.^12^ This is particularly the case since the COVID-19 pandemic as community pharmacists have continued to provide face-to-face support and have been involved in administering the COVID-19 vaccine.^13^^,^^14^ As a result of the greater demands on the NHS since the COVID-19 pandemic, community pharmacists’ roles are to be expanded further to reduce the burden on general practice. NHS England’s *Delivery Plan for Recovering Access to Primary Care* outlines how oral contraceptives and blood pressure checks will be expanded across community pharmacy services.^15^

**Table:** How this fits in

Community pharmacies are well placed to provide and facilitate access to care for those most disadvantaged in society. This is particularly the case since the COVID-19 pandemic as they have continued to provide face-to-face support whereas other primary care services have seen a rapid transformation to digital services and remote consultations. This study highlights that further resources, such as access to translation services, and interventions to enable better communication between primary care providers and community pharmacy teams are needed to further utilise the skills of community pharmacists.

Although community pharmacy teams may be well placed to work with general practices to reduce health inequalities, little is known about their contributory role. Against this backdrop, this study aimed to explore the experiences and needs of community pharmacy teams in providing care for people from marginalised groups with a focus on how their role has changed since the COVID-19 pandemic.

## Method

### Design

A qualitative study based on semi-structured interviews was undertaken to explore the role of community pharmacy teams in providing or facilitating care for marginalised communities.

### Participants and recruitment

The participants predominantly comprised of staff working in community pharmacies (for example, pharmacists and accredited, dispensing assistants). Organisational structures, policy, and practice can have a significant impact on health inequalities,^16^ therefore a subsample of GPs, practice nurses, and primary care network (PCN) pharmacists were also included. Clinical pharmacists are a key member of multidisciplinary teams in PCNs that were developed, in part, to improve integrated working across the NHS. PCN pharmacists will often work and support general practices with medication reviews and prescriptions.^17^^,^^18^ By interviewing a subsample of general practice and PCN staff, the aim was to explore collaborative working across different organisational structures and within the boundaries of different policies and practices.

Contact details of community pharmacies and general practice staff were obtained from online searches, with support from the Greater Manchester Clinical Research Network (CRN) and professional networks. Participants were approached to participate in the research via email and sent a participant information sheet. Community pharmacies located in the North of England in areas with deprivation scores <5^19^ were approached; however, as the authors advertised for participants via the CRN and online, some pharmacists volunteered to take part on the basis that they felt they served other marginalised communities.

### Data collection

Data collection took place between March and August 2022. Participants were invited to participate in a semi-structured interview and were given the option of a telephone or face-to-face interview. In total, 31 professionals took part in an interview: 21 community pharmacy staff, five PCN pharmacists, three GPs, and two nurses. Seven interviews took place face-to-face and the remainder took place via the telephone. Interviews ranged in length from 17–61 min.

A topic guide for community and PCN pharmacists was developed and informed by the literature and subsequently reviewed and discussed with a community pharmacy participation and involvement group based in the Patient Safety Translational Centre at The University of Manchester, which consists of seven community pharmacists working across Greater Manchester. The authors met with this group at various time points throughout the project, including protocol development and analysis to gain their expertise and advice on recruitment and interview content. The topic guide for GPs and nurses differed slightly, and although similar themes were discussed, the interviews focused on how and if they worked with community and PCN pharmacists to deliver care for these groups. All interviews were audiorecorded and participants completed a consent form before the interview. Participants were also reimbursed personally for their time and were paid by the rates suggested by the Greater Manchester CRN.

### Data analysis

The audiorecorded interviews were transcribed verbatim and analysed using an adapted framework method approach.^20^ This approach allows for analysis according to predefined themes and themes that emerge more inductively from the data. The analytical framework was completed in NVivo (version 12) and guided by the questions posed in the interview schedule and the literature. To increase rigour, initial transcripts were coded independently by two researchers (the first author and the senior author) and interpretation of the data was discussed in regular team meetings to develop and refine core themes. The results were also discussed with the community pharmacy participation and involvement group to gain their insights on the main themes and recommendations for future research.

## Results

### Sample characteristics

Of the 31 participants, 16 were community pharmacists, five were PCN pharmacists, and five worked in other roles within community pharmacy (dispensers — qualified and trainee). Interviews were also completed with three GPs and two practice nurses. Seventeen participants were male and 14 were female. Eleven participants worked in independent community pharmacies; nine in ‘chain’ community pharmacies and two were locum community pharmacists. All participants worked in the North of England. See Box 1 for participant characteristics.

**Box 1.** Participant characteristics

**Participant number**	**Gender**	**Role**	**Type of pharmacy**
1	Male	Community pharmacist	Medium chain
2	Female	Community pharmacist	Independent
3	Male	Community pharmacist	Independent
4	Male	Community pharmacist	Independent
5	Male	Community pharmacist	Independent
6	Male	Locum community pharmacist	N/A
7	Male	Community pharmacist	Independent
8	Female	Dispenser	Independent
9	Male	Community pharmacist	Independent
10	Female	Pre-registration community pharmacist	Independent
11	Female	Dispenser	Independent
12	Male	PCN pharmacist	N/A
13	Female	Locum community pharmacist	N/A
14	Male	Community pharmacist	Medium chain
15	Female	Community pharmacist	Medium chain
16	Male	Community pharmacist	Large chain
17	Female	Dispenser	Large chain
18	Female	PCN pharmacist	N/A
19	Male	Community pharmacist	Independent
20	Male	Community pharmacist	Independent
21	Female	PCN pharmacist	N/A
22	Male	PCN pharmacist	N/A
23	Female	PCN pharmacist	N/A
24	Female	Trainee dispenser	Small chain
25	Female	Trainee dispenser	Small chain
26	Male	Community pharmacist	Small chain
GP1	Male	GP	N/A
GP2	Male	GP	N/A
GP3	Male	GP	N/A
N1	Female	Advanced nurse practitioner	N/A
N2	Female	Advanced nurse practitioner	N/A

*N = nurse. N/A = not applicable. PCN = primary care network.*

### Findings

The findings are presented under two key themes: defining and identifying patients who are marginalised; and delivering care to patients who are marginalised. Five subthemes relating to knowledge and training; continuity of care; the wider primary care context: remote consultations and digital inequality; access to translation services; and integrated working are also presented.

### Defining and identifying patients who are marginalised

#### Knowledge and training

Pharmacy participants were asked what marginalised groups they worked with in their local area and how they identified them. Definitions varied depending on the demographics of the local population. Where pharmacy staff had worked in a locality for a significant period of time they described familiarity with individuals that enabled them to identify those from marginalised groups. Another means by which pharmacy teams were able to identify individuals from marginalised groups was by the information contained within the prescription or on the pharmacy’s computer system:

*‘The people with addiction problems normally go on a programme so they have certain prescriptions, so you know from that. And then sometimes you know someone’s homeless from the description, sometimes the doctor has a note, or their address is the doctors’ address.’*

(Participant 13: locum community pharmacist, 6 years’ experience)

*‘We do have a section on our computer, yes. If we search for patients, we know if they’re transgender or there is a little box, kind of, thing that can say like vulnerable or categorises them slightly, yes.’*

(Participant 17: dispenser, 1 year of experience)

Frequently, community pharmacy teams relied on visual and audio clues to identify individuals from marginalised groups and included references to their age, ethnic origin, language, or appearance:

*‘I’ll use Google Translate on my phone which is really handy nowadays, you can just take it and just speak into it and they can speak into it and get information across that way, and often that’ll be the way that we find out maybe that someone is an asylum seeker.’*

(Participant 6: locum community pharmacist, 6 years’ experience)

*‘There is another like a women’s refuge. These are people who are maybe affected with domestic abuse or any kind of abuse. You can tell by their dress that this lady is from there. You get different groups of people coming here. You can just recognise by their dress.’*

(Participant 7: community pharmacist, 15 years experience)

Although the majority of pharmacy teams interviewed had not received any formal training on working with marginalised communities there was an awareness, particularly during and since the pandemic, that working together as a team was essential in supporting marginalised groups to access and receive care:

*‘I think it’s my experience and our experience as a team because we’re a great team. I think it’s the fact that we have just accepted that we were going to have to deal with it more and we’re going to have to go that extra mile. Because if we didn’t, who are going to?’*

(Participant 2: community pharmacist, 25 years’ experience)

There was a lack of awareness as to whether specific training was available; however, there was also scepticism as to whether training was possible given continued workload demands and time pressures. There was also apprehension as to whether training would be necessary or relevant. For example, one participant suggested that working with marginalised groups was ‘instinctual’ while another did not see the need for training as all patients were treated the same:

*‘When they come in* [LGBTQ+] *it is like we treat them exactly the same, so we never thought there should be any training.’*

(Participant 7: community pharmacist, 15 years’ experience)

When faced with questions from individuals from marginalised groups that pharmacy staff were unable to address, staff were often able to signpost to other services or sources of support. Participants frequently mentioned having leaflets and posters on display in the pharmacy and in situations where they were unsure how to help, many pharmacy staff were proactive in trying to find out:

*‘We need to always think about the patient themselves … If they need any other help, we can always research from our end, if there are any local providers.’*

(Participant 19: community pharmacist, 7 years’ experience)

### Delivering care to patients who are marginalised

#### Continuity of care

Continuity of care was an important factor in enabling community pharmacists to both identify and facilitate care for marginalised groups. Participants were not explicitly asked about continuity of care during interviews, nor did any participants use the phrase. It was felt by some participants that continuity enabled individuals to feel comfortable to approach their pharmacist about sensitive health or social issues:

*‘I think many people from marginalised society or community, they feel more comfortable to come to the pharmacy and ask questions, than GPs or other healthcare sectors. Because we serve them more often. And we have some patients for years now. So, when we serve them, we talk to them … And I think they are more comfortable to come to us and speak up.’* (Participant 10: pre-registration community pharmacist, newly-qualified, 1st year)

This was particularly the case with community pharmacies that worked with drug and alcohol addiction or people experiencing homelessness as daily attendance at the pharmacy helped to facilitate care in some instances:

*‘Some of my experiences with people who are homeless, they tend, or can be quite suspicious of, either their key worker or GPs but just through them coming to the pharmacy, day in, day out, you do get a bit more of a relationship with them.’*

(Participant 9: community pharmacist, 9 years’ experience)

#### The wider primary care context: remote consultations and digital inequality

Digital access issues combined with limited knowledge and trust in the remote system resulted in some individuals accessing care via their community pharmacy. Pharmacy participants acknowledged that since the start of the COVID-19 pandemic there had been increased footfall, with some community pharmacy staff suggesting there were insufficient GPs to meet patient demand, which increased their workload:

*‘Often you’re only getting telephone consultations with doctors, and that can be quite difficult for patients that can’t articulate things very well … whether there’s a language barrier there, yes … but it might not just be about the language … you’ve also got the option to send doctors pictures and things … but not everyone can do that … not everyone’s got the internet connection to do that … Not everyone’s got a phone that can take a picture and not everyone knows how to send an email to the doctors, so there’s a lot of new barriers that are now there that weren’t there before, and that does seem to affect the less advantaged groups.’*

(Participant 6: locum community pharmacist, 6 years’ experience)

Signposting was not always considered sufficient, with some pharmacy team members also facilitating access to care. There was a sense that facilitating care was not necessarily an ‘expected’ part of their role but something that many community pharmacists did to ensure the care needed was received, particularly since the pandemic:

*‘I’ve booked a lot of elderly patients COVID tests, so they get a text message and it sends a link and they’re just not sure how to do it … Last week I took a picture of a patient’s … they had a growth on their hand, took some high-quality pictures and emailed it to the doctors for them in preparation for their appointment because she had a flip phone with a terrible camera.’*

(Participant 6: locum community pharmacist, 6 years’ experience)

#### Access to translation services

Some pharmacists highlighted that medication safety was particularly important for some marginalised groups, particularly people who did not speak English and people with dementia and/or mental health issues, as they were sometimes prescribed medication that they did not understand how to take or why the medication had been prescribed:

*‘I think if it’s to do with medications and things, often I think the education about how to use medicines and when to use them and how to get them, those kind of questions are asked more often by people that are seeking asylum. They sometimes don’t have the knowledge about … some of the basic medications you know all about generally if you’re born in the UK.’*

(Participant 6: locum community pharmacist, 6 years’ experience)

Communicating with individuals with limited English was frequently mentioned as a barrier to providing care. Unlike GPs, pharmacy staff did not have access to translation services, which often meant they relied on ‘Google Translate’ via their smartphone:

*‘Google Translate, yeah, it helps from time to time, it often is pretty accurate. There may be something like another translation software that might work out a little bit better, like one that can actually maybe record speech specifically.’*

(Participant 24: trainee dispenser, 2 months’ experience)

There were safety concerns regarding this approach among some participants who questioned the accuracy of Google Translate. Sometimes pharmacists relied on patients’ relatives or friends instead to translate for them, but they felt that this was not always appropriate, particularly when this relative was a child. There was also concerns that some individuals did not understand why they had been prescribed something, leading to pharmacists having to discuss their diagnosis in addition to the medication.

#### Integrated working

In situations where pharmacies needed to liaise with GPs over patient care, the most commonly reported method was via telephone. However, this was extremely problematic as more often than not the pharmacists found themselves waiting in the GP’s telephone queuing system. Emails were used on occasion; however, the length of time often taken for the GP to respond was also cited by participants as problematic:

*‘With the GPs, you know, the seven or eight that we deal with, we don’t have private phone numbers, so we, kind of, have to just sit in line on their main switchboard and just wait, or I tend to normally send them an email, and a few days later I’ll get a phone call back. But if anything is urgent, it’s hard because we haven’t got the time to spend on hold to them, when the patient is in the shop, huffing and puffing.’*

(Participant 1: community pharmacist, 3 years’ experience)

Where PCN pharmacists were located in general practices this was viewed positively by community pharmacists and GPs alike in improving the relationship between pharmacies and GPs. This was often because community pharmacists could direct any queries via email or telephone directly to the PCN pharmacist without having to go via GP receptionists. The location of the pharmacy and access to a direct telephone line were also important factors, with some pharmacy teams recognising a valuable relationship with a nearby practice whereby they could ‘drop by’. In instances where pharmacies were located central to a number of practices, participants sometimes reported struggling to form working relationships:

*‘I think one of the key factors is the working relationship with the GPs. But if you can be friendly, and if they trust your judgement and you trust theirs, then you can work very collaboratively, and it would definitely benefit the patient, no question.’*

(Participant 20: community pharmacist, 44 years’ experience)

There was recognition from the GPs interviewed that relationships with pharmacists, although useful in providing care to marginalised groups, were often varied and dependent on the community pharmacy in question:

*‘Some pharmacists are amazing and some pharmacists are very difficult to work with and we try to bring things up and it’s repeated things that we just … we say to them and then we have to keep calling them up and saying things and change things and it’s … and it really depends on the pharmacy.’*

(Participant GP1: GP, 15 years’ experience)

It is worth noting, however, that the nurses interviewed in this study had little or no experience of working with community pharmacists. The authors struggled to recruit practice nurses to be involved in the study as they felt they could not contribute to the study as they had little or no interactions with community pharmacists.

Pharmacy participants discussed how positive working relationships with a variety of health and social care services enabled them to provide further support to marginalised groups. This included community groups; district nurses; drug teams; nursing homes; PCNs; social services; rough sleeper teams; safeguarding teams; and social prescribers:

*‘So, the staff at the charity office, which is not too far, would bring service users here. And they would, if they’ve got any medical issue or any question regarding drugs or anything, they will ask me.’*

(Participant 5: community pharmacist, 42 years’ experience)

There was recognition from the GPs interviewed that working with community pharmacists was important for facilitating care to marginalised groups, but until the COVID-19 pandemic, their capabilities had perhaps been undervalued. As one GP alluded to, the pandemic enlightened other primary care services as to the breadth of services offered by community pharmacy teams:

*‘I mean I believe that we’re underusing what community pharmacists could do. During the pandemic it showed us that they could have been doing so much more. They could be seen as part of the primary care offer rather than a place to just collect your ’scripts, you know?’*

(Participant GP1: GP, 15 years’ experience)

## Discussion

### Summary

This qualitative study aimed to explore the experiences and needs of community pharmacy teams in providing care for people from marginalised groups with a focus on how their role has changed since the COVID-19 pandemic. The rapid transformation to digital services and remote consultations in primary care accelerated by COVID-19^21^^,^^22^ has had unintended consequences for the workload of community pharmacy teams. Community pharmacy teams have been pivotal in advocating on behalf of marginalised groups who perhaps feel left behind by the transition to digital health care. Although community pharmacy teams generally felt they had the skills and experience to provide care for marginalised groups, lack of access to translation services was considered a key barrier to providing care, with many community pharmacy teams relying on smart phones to communicate with their patients. As the majority of participants worked in areas with high levels of deprivation, they perhaps faced greater challenges as a result of the increased level of support some patients needed during COVID-19 because of their limited knowledge of or access to digital technology.

An integrated approach was often difficult owing to limited means of communication between GPs and community pharmacy teams making it difficult for providers to work together. PCN pharmacists working in primary care, geographical proximity, and direct phone numbers to bypass patient queries were considered helpful in facilitating this collaborative relationship; often, however, community pharmacists were working in isolation. The findings highlight that the success of providing care for marginalised groups relies heavily on relationship continuity with patients, and ingenuity and proactivity from community pharmacy teams working together to provide and facilitate this. More flexible and collaborative working across health and community organisations, in addition to access to translation services, would be advantageous in enabling community pharmacists to provide access to safer and equal care for marginalised communities.

### Strengths and limitations

This study captured a wide range of diverse experiences by engaging community pharmacy teams across different pharmacies in the North of England. Although these findings will not necessarily be representative of all community pharmacies, and it is recognised there may be organisational differences between community pharmacies in the independent and multiple sectors that this study has not been able to capture, a strength of this study is that it captured rich data that revealed some key barriers to providing care for marginalised groups in the community pharmacy sector. The use of semi-structured interviews meant that the data captured what participants said they did. Using additional methods such as observation would have enabled the capturing of what participants actually do, thus strengthening the findings.

### Comparison with existing literature

To the authors’ knowledge, this is one of the first studies to explore the role of community pharmacy teams in providing care for marginalised groups. Much of the existing literature focuses on how the physical accessibility of community pharmacies may mitigate the inverse care law^23^ for people living in deprived areas.^7^ However, although convenience and locality may be an important factor in how marginalised communities engage with health care, definitions of access are complex and other factors, such as disease burden, expectations, attitudes, and knowledge of services, may also result in an underutilisation of healthcare services by those who need them most.^24^ Organisational structures, policy regulations, and medical resources can also limit access to care.^25^^,^^26^ This finding adds to recent research that shows that although community pharmacies are more physically accessible than general practices, developing a trusting relationship with a pharmacist is an important factor influencing persistent engagement with health care.^8^ Although research on the transformation to remote primary care during and following COVID-19 is still in its infancy, it has, to date, focused on general practice services^21^ rather than wider implications for community-based services such as pharmacy.

The NHS Long Term Plan advocates a *‘collaborative’* approach to reducing health inequalities and from July 2022 introduced a new organisational structure across the NHS (integrated care boards or systems) made up of multiple health and social care organisations working together; however, previous research shows that despite common interests, pharmacists and GPs often work in isolation from each other.^27^^–^^29^ There is evidence of organisational barriers preventing the integration of general practices and community pharmacies, and the current study highlighted one such barrier in the form of direct lines of communication between community pharmacies and GP practices. PCN pharmacists can act as a conduit of communication; however, not all general practices have a PCN pharmacist working on site full-time to enable timely interactions across services. As patients from marginalised communities often present with more complex health needs, easy and accessible lines of communication are essential if they are to work together to facilitate access to care and reduce inequalities.

With the increase in international migration to the UK over the past 20 years, it is well recognised that language barriers have led to poor-quality care and health outcomes.^30^ Although the evidence suggests that the implementation of translation services is beneficial and would improve patient access and health outcomes,^30^^,^^31^ research on how patients view and use these services is limited. Further, current evidence suggests these services are often underused, with a number of factors such as fear of putting financial strain on the NHS and the preferences of both patients and health professionals to rely on friends and family being critical.^30^^,^^32^^,^^33^ Whitaker *et al* found that the commissioning of translation services was inconsistent across primary care in England and Wales,^30^ with the current research highlighting that interpreting services are not generally commissioned for community pharmacies. The use of Google Translate was prevalent within the current study, which supports the literature regarding the need for translation services across all health services to enable safe and equal care for people whose first language is not English. However, in the absence of translation services, caution should be exercised when using Google Translate as a substitute as its accuracy varies across languages^34^ and in particular its accuracy when translating medical phrases.^35^

### Implications for research and practice

In the context of a post-pandemic society in which remote care and digital technologies are increasingly relied on to navigate healthcare systems, the roles of community pharmacy teams are increasingly important. There are opportunities to utilise the skills of community pharmacists, but further resources are needed. Although the community pharmacy teams in the current study were undecided about the benefits of training, as part of the pharmacy contract (the agreement that exists between the NHS and community pharmacy services) community pharmacists will be asked to complete training on health inequalities, in addition to specific training on reducing domestic violence. Based on the empirical evidence, the authors suggest that other resources, such as access to translation services and interventions to enable better communication between primary care providers and community pharmacy teams, should be considered. Interventions such as a direct telephone line to connect general practice and community pharmacies could be one way of facilitating a closer working relationship. A more detailed understanding of how translation services are commissioned and how they could be used in community pharmacies is a crucial next step to improve access to health care for some marginalised groups.
